# Muskuloskelettale Labordiagnostik im Leistungssport

**DOI:** 10.1007/s00132-021-04072-1

**Published:** 2021-02-22

**Authors:** Maximilian M. Delsmann, Julian Stürznickel, Michael Amling, Peter Ueblacker, Tim Rolvien

**Affiliations:** 1 Institut für Osteologie und Biomechanik, Universitätsklinikum Hamburg-Eppendorf, Hamburg, Deutschland; 2 Praxis für Orthopädie und Sportmedizin, München, Deutschland; 3 Fachbereich Orthopädie, Klinik und Poliklinik für Unfallchirurgie und Orthopädie, Universitätsklinikum Hamburg-Eppendorf, Martinistraße 52, 20246 Hamburg, Deutschland

**Keywords:** Biomarker, Professionelle Athleten, Regeneration, Stressfraktur, Vitamin D, Biomarker, Professional athletes, Regeneration, Stress fractures, Vitamin D

## Abstract

**Hintergrund:**

Die labordiagnostische Untersuchung stellt eine wichtige Möglichkeit zur Beurteilung und Optimierung der Leistungs- und Regenerationsfähigkeit professioneller Athleten dar. Ferner ist sie für die Prävention, Diagnostik und Rehabilitation von Verletzungen und Überbelastungen von Bedeutung.

**Fragestellung:**

Ziel dieser Arbeit ist die Darstellung muskuloskelettaler laborchemischer Parameter, die relevante Erkenntnisse für die medizinische Betreuung von Leistungssportlern liefern.

**Material und Methoden:**

Literaturrecherche und narratives Review.

**Ergebnisse:**

Die Bestimmung des Vitamin-D-, Calcium- und Knochenstoffwechsels stellt die laborchemische Basisdiagnostik im Rahmen der Beurteilung des Skelettstatus mit zusätzlichem präventivem Nutzen bezüglich muskuloskelettaler Verletzungen dar. Ferner können muskuläre Serummarker, z. B. Laktatdehydrogenase (LDH), Kreatinkinase (CK), Myoglobin und Aspartat-Aminotransferase (ASAT), helfen, eine metabolische Adaptation an das physische Training festzustellen und Aussagen über die muskuläre Arbeitslast und mögliche Schädigungen zu gewinnen. Die Energieverfügbarkeit kann durch eine entsprechende Bilanzierung sowie die laborchemische Bestimmung der Makro- und Mikronährstoffe eingeschätzt und optimiert werden.

**Schlussfolgerungen:**

Die labordiagnostische Untersuchung besitzt in der Betreuung von Athleten eine sportartenübergreifende klinische Relevanz. Sie dient der Erreichung einer höchstmöglichen Leistungsfähigkeit sowie optimalen Prävention von Knochen- und Muskelverletzungen, wobei sämtliche Mangelzustände (z. B. Vitamin D) ausgeglichen werden sollten. Durch eine Periodisierung der laborchemischen Untersuchungen, mit zumindest zwei Labordiagnostiken im Jahr, und Aufstellung individueller Variabilitäts- und Referenzbereiche kann ferner eine bessere Beurteilbarkeit erreicht werden.

## Abstract (en)

**Background:**

Laboratory diagnostics represent a valuable tool for the optimization and assessment of the performance and regeneration ability in professional athletes. Blood parameters play an important role in the prevention, diagnosis and rehabilitation of injuries and physical overload.

**Objectives:**

The aim of this article is to present an overview of musculoskeletal laboratory parameters and to provide relevant information for the medical care of competitive athletes.

**Methods:**

Literature search and narrative review.

**Results:**

The laboratory assessment of bone metabolism includes vitamin D, calcium and bone turnover and aims to provide a preventive benefit with respect to skeletal complications (e.g., to minimize the risk of bone stress injuries). In addition, muscular serum markers, such as lactate dehydrogenase (LDH), creatine kinase (CK), myoglobin and aspartate aminotransferase (AST) can be used to monitor metabolic adaptation to physical exercise and to obtain information about the muscular workload and potential damage. The energy availability can be estimated and optimized by appropriate balancing and laboratory determination of macro- and micronutrients.

**Conclusions:**

Laboratory diagnostics have a clinical relevance across different sport disciplines. They are intended to support athletes and medical staff on their way to the highest possible performance and help to ensure the optimal prevention of bone and muscle injuries. Parameters with deficiency results (e.g., vitamin D) should be adequately compensated. A periodization of the laboratory tests, with at least two tests per year, and the establishment of individual variability and reference ranges can improve the assessment.

Die Labordiagnostik stellt in der medizinischen Betreuung von Leistungssportlern eine wichtige diagnostische Säule dar. Dabei können mithilfe geeigneter Laborparameter essenzielle Informationen zur Beurteilung der Leistungs- und Regenerationsfähigkeit von Athleten gewonnen werden. Weiterhin ist die Labordiagnostik sowohl in der Prävention als auch in der Diagnostik und Rehabilitation von Verletzungen von Bedeutung. Insbesondere liegt der Fokus auf dem Erkennen und Ausgleich von Mangelzuständen. Diesem Präventionsgedanken muss angesichts der immer größer werdenden Trainings- und Wettkampfbelastungen im Spitzensport eine hohe Bedeutsamkeit zugeschrieben werden.

Die verschiedenen im Blut messbaren Biomarker liefern reproduzierbare, objektive, wertvolle und valide Informationen über biologische Prozesse und Krankheitszustände. Neben den klassischen hämatologischen Werten werden zunehmend auch muskuloskelettale Biomarker zur Analyse genutzt, welche adäquat mess- und analysierbar sind und Hinweise auf sowohl physiologische als auch pathologische Prozesse des muskuloskelettalen Systems bieten. Ziel dieser Arbeit ist die Übersicht einer praxisrelevanten muskuloskelettalen Labordiagnostik, welche relevante Informationen für die ärztliche Betreuung von Leistungs- und Spitzensportler liefern soll.

Die Erkenntnisse sollen Athleten und Betreuer auf dem Weg zu einer höchstmöglichen Leistungsfähigkeit und bestmöglichen Prävention von Verletzungen unterstützen. Beispielsweise konnte die klinische Relevanz einer strukturiert durchgeführten Labordiagnostik bei männlichen Athleten einer professionellen amerikanischen Sportmannschaft aufgezeigt werden, welche über 9 Jahre im Rahmen der Saisonvorbereitung durchgeführt wurde. Bei 10 % der Athleten wurden pathologische Laborwerte detektiert, welche wiederum bei 40 % weiterführende Laboruntersuchungen erforderlich machten [1]. Die vorliegende Arbeit liefert einen Überblick über die wichtigsten laborchemischen Parameter in der muskuloskelettalen Diagnostik hinsichtlich 1) des Knochenstoffwechsels, 2) des Muskelstatus und 3) der Ernährung (Abb. 1). Obwohl Verletzungen der Sehnen (Tendinopathien, Enthesiopathien und Rupturen) eine große Rolle bei Athleten spielen [2] und die Labordiagnostik diesbezüglich einen wissenschaftlichen Stellenwert hat, spielt sie jedoch in der klinischen Diagnostik aktuell eine untergeordnete Rolle und wird somit in diesem Artikel nicht beleuchtet.

**Figure 1:**
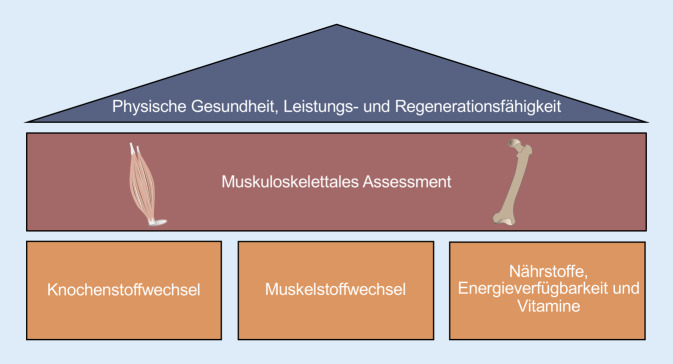

## Knochenstoffwechsel

Für Leistungssportler mit hohen körperlichen Belastungen ist ein intakter Knochenstoffwechsel von essenzieller Bedeutung. Der Knochen bildet als Endoskelett neben der Muskulatur, den Sehnen und Bändern das Hauptorgan des Bewegungsapparates. Er garantiert die Beweglichkeit und Stabilität und unterliegt ständiger Adaptation. Mittels labordiagnostischer Parameter, welche sich als Marker des Knochenumbaus („remodelings“) und der Mineralstoffhomöostase eignen, lässt sich neben apparativen diagnostischen Methoden der metabolische Knochenstatus des Athleten abbilden [3].

Die Knochenmineralisation wird maßgeblich über die Calciumhomöostase reguliert. Die empfohlene Tagesaufnahme von Calcium beträgt 1000–1500 mg/Tag [4, 5]. Neben Calcium ist Phosphat als Mineralstoff ebenfalls maßgeblich an der Mineralisation des Knochens beteiligt. Bei nicht ausreichender Versorgung des Körpers mit Calcium und/oder Phosphat resultiert eine reduzierte Knochenmineralisation bis hin zur Osteomalazie, begleitet von einer muskulären Insuffizienz [6, 7]. Chronische Hypophosphatämien können vielfältige Ursachen haben und entstehen beispielsweise durch Mangelernährung (v. a. Anorexia nervosa), in Zusammenhang mit Vitamin-D-Mangel, bei genetischen Erkrankungen (v. a. Phosphatdiabetes), paraneoplastisch (v. a. onkogene Osteomalazie) oder medikamentenassoziiert (u. a. Antazida, Diuretika) [6].

Von übergeordneter Bedeutung für den Knochenmetabolismus und die Knochenfestigkeit sind das Steroidhormon Calcitriol (1,25(OH)_2_-Vitamin‑D_3_), das Parathormon (PTH) sowie der „fibroblast growth factor“ 23 (FGF23), welche die Hauptregulatoren der Calcium- und Phosphathomöostase darstellen [8–10]. Calcitriol steigert als aktiver Metabolit des Vitamin D_3_ (Cholecalciferol) die intestinale und renale Resorption von Calcium, erhöht somit die Calciumkonzentration im Körper und führt zu einer Mineralisation unmineralisierter Knochensubstanz (Osteoid) [11]. Bei einem Vitamin-D-Mangel und konsekutiver enteraler Calciumaufnahmestörung kann durch eine kompensatorisch gesteigerte PTH-Sekretion (sekundärer Hyperparathyreoidismus) eine Stimulation der knochenresorbierenden Osteoklasten und somit eine vermehrte Calciummobilisation aus dem Knochen induziert werden. Dies findet auf Kosten der Knochenqualität statt und kann durch eine Reduktion des Knochenmineralsalzgehaltes und der Knochenstruktur zu einer Verschlechterung der Knochenstabilität führen [12]. Ein weiterer häufiger Grund für eine gestörte enterale Calciumaufnahme ist die (chronische) Einnahme von Protonenpumpeninhibitoren mit konsekutiver Hypochlorhydrie [13]. Ferner wird in diesem Zustand die renale Phosphatelimination gesteigert, da ein erhöhter Phosphatspiegel wiederum einem Calciumanstieg entgegenwirken würde. Eine erhöhte PTH-Konzentration bei gleichzeitig erhöhtem Calciumspiegel spricht hingegen für eine autonome Überfunktion der Nebenschilddrüse im Sinne eines primären oder tertiären (Folge einer chronischen Überstimulation nach sekundärem) Hyperparathyreoidismus [9]. FGF23 ist ein endokrines Hormon, welches eine Phosphaturie in der Niere verursacht, während die Produktion von Calcitriol gehemmt wird [10]. Die laborchemische Bestimmung von FGF23 stellt aktuell noch keine Routinediagnostik dar, ist jedoch bei rezidivierenden Hypophosphatämien und Verdacht auf eine genetische oder erworbene Phosphatstoffwechselstörung indiziert.

Die klinische Relevanz von Vitamin D ist in der sportmedizinischen Betreuung von Leistungs- und auch Gelegenheitssportlern hoch, da Vitamin D aufgrund einer direkten oder indirekten Beeinflussung der Leistungs- und Regenerationsfähigkeit sowie des Verletzungsrisikos, wie für ossäre Stressreaktionen, einen maßgeblichen Effekt auf die Gesundheit des Athleten haben kann [14–16]. Dennoch stellt ein Vitamin-D-Mangel im professionellen Sport ein häufig auftretendes Phänomen dar [1, 17–20]. So konnte bei 13 von 20 männlichen Profifußballern des englischen Premier-League-Teams Liverpool FC im Dezember 2010 ein insuffizienter Vitamin-D-Status erhoben werden [17]. Auch bei 31 von 70 männlichen Handballspielern aus der ersten deutschen Handballliga konnte ein Vitamin-D-Mangel festgestellt werden [18]. Dieser Mangel ist nicht nur ausschließlich in den Wintermonaten oder bei Hallensportlern, sondern über das ganze Jahr hinweg und auch bei anderen Sportarten zu beobachten. So zeigten lediglich 25 von 80 Footballspielern der National Football League (NFL) im Rahmen der routinemäßigen Gesundheitsuntersuchungen während der Saisonpause und in der Vorbereitungsphase adäquate Vitamin-D-Spiegel, wobei das Risiko für einen Vitamin-D-Mangel bei dunkelhäutigen Athleten erhöht war [19]. Hierbei ist zu bedenken, dass eine derart hohe Prävalenz an Mangelzuständen trotz in den Vereinigten Staaten teilweise mit Vitamin D angereicherter Lebensmittel vorlag [21]. Ein optimaler Referenzwert für Vitamin D bei Leistungssportlern ist Gegenstand von Diskussionen [7, 14, 15, 22, 23]. Zur Beurteilung, ob ein Athlet oder eine Athletin ausreichend mit Vitamin D versorgt ist, wird in der Regel das Calcidiol (25(OH)-Vitamin-D3) im Serum gemessen. Zunächst konnte auf histologischer Ebene in der Allgemeinbevölkerung gezeigt werden, dass ein Vitamin-D-Spiegel (25(OH)D_3_) von ≥ 30 ng/ml bei Frauen und Männern eine Hypomineralisation im Sinne einer Osteomalazie weitestgehend ausschließt [7]. Während bei Vitamin-D-Konzentrationen von ≥ 40 ng/ml von einem präventiven Nutzen bezüglich (Stress‑)Frakturen auszugehen ist, scheint ein Spiegel von ≥ 50 ng/ml für beide Geschlechter eine optimale Voraussetzung für eine maximale Leistungsfähigkeit der Athleten darzustellen [22]. So konnten Williams und Kollegen in verschiedenen amerikanischen Männer- und Frauenprofiteams (Crosslauf, Basketball, Fußball, Leichtathletik) zeigen, dass durch eine 8‑wöchige Vitamin-D-Supplementation mit 50.000 IE pro Woche bei Vitamin-D-insuffizienten Athleten (<30 ng/ml) eine Reduktion der Inzidenz von Stressfrakturen von 7,5 % auf 1,6 % erzielt werden konnte [14]. Auch bei 5201 untersuchten weiblichen Navy-Rekruten konnte unter Calcium- (2000 mg/Tag) und Vitamin-D-Supplementation (800 IE/Tag) eine um 21 % geringere Inzidenz für Stressfrakturen festgestellt werden [16]. Umgekehrt war in einem Kollektiv von 53 Patienten mit Stressfrakturen bei 44 Betroffenen (83 %) ein Vitamin-D-Spiegel von < 40 ng/ml zu beobachten [15].

**Figure 2:**
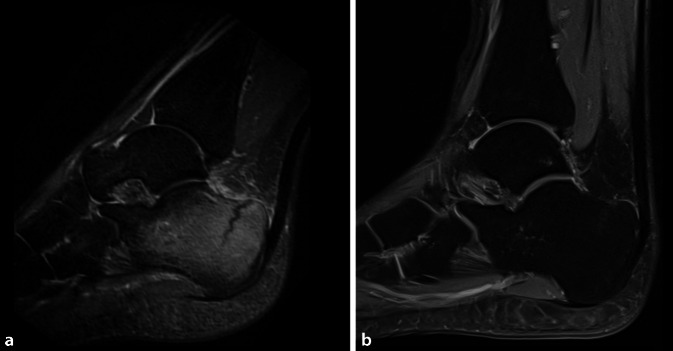

Auch was die Rekonvaleszenz nach eingetretener Fraktur anbetrifft, scheint Vitamin D eine entscheidende Rolle zu spielen. In einem Kollektiv von 617 Patienten beider Geschlechter konnte gezeigt werden, dass sich die Inzidenz einer verzögerten Frakturheilung („delayed union“) bei Patienten mit Vitamin-D-Mangel (9,7 %) signifikant von denen mit einem suffizienten Vitamin-D-Spiegel (0,3 %) unterschied [23]. Bei Patienten mit initialem Mangel und anschließender Supplementation von 1200 IE Vitamin D täglich über 4 Monate konnte eine „delayed union“ ebenfalls signifikant reduziert werden [23]. Dieses verdeutlicht eindrücklich, dass selbst bei einer Vitamin-D-Insuffizienz zum Zeitpunkt einer Fraktur ein Ausgleich dieses Defizits von großem klinischem Nutzen sein kann. Die Bedeutung eines ausgeglichenen Vitamin-D-Spiegels und einer balancierten Calciumhomöostase bezüglich der klinischen und radiologischen Entwicklung von Stressfrakturen bei Leistungssportlern kann im angeführten Fallbeispiel verdeutlicht werden, in dem bei einer Marathonläuferin nach konsequenter Anpassung der Belastung und einer adäquaten Vitamin-D-Supplementation eine Ausheilung der Stressfraktur erreicht werden konnte (Abb. 2).

Um die Balance des Knochenstoffwechsels beurteilen zu können, werden laborchemische Knochenformations- und Knochenresorptionsmarker herangezogen. Ihre Messwerte spiegeln das Ausmaß des Knochenaufbaus bzw. -abbaus wider. Formationsmarker stellen unter anderem die knochenspezifische alkalische Phosphatase („bone-specific alkaline phosphatase“ [BAP]) und das Osteocalcin (Oc) dar. Die BAP gehört als enzymatisches Isoenzym der ubiquitär vorkommenden Gesamt-AP an. Die Bestimmung der BAP wird der Gesamt-AP aufgrund der höheren Spezifität vorgezogen. Sie ist an allen Phasen der Knochenmineralisation beteiligt und kann als Indikator für die Osteoblastenaktivität und die Knochenformation fungieren [24]. Osteocalcin ist ein Hydroxylapatit-bindendes Protein, welches als spezifischer Marker für Osteoblasten gilt und eine maßgebliche Rolle bei der Regulierung der Knochenmineralisation spielt [25].

Es gibt jedoch auch Evidenz, dass Osteocalcin eine hormonelle Rolle, beispielsweise in der Regulation des Energiestoffwechsels sowie der Fertilität, einnimmt [26, 27]. Durch das Fehlen einer methodischen Standardisierung und der geringen Halbwertszeit variieren die Werte zum Teil stark zwischen verschiedenen Laboren, was die Aussagekraft einschränken kann [24]. Eine laborchemische Dissoziation zwischen erhöhter beziehungsweise hoch normaler BAP bei Vorliegen eines in Relation deutlich niedrigeren Osteocalcins spricht für das Vorliegen einer osteomalazischen Komponente, das heißt einer Mineralisationsstörung des Knochens. Durch das Auftreten höherer Osteocalcin- und BAP-Werte bei Triathleten im Vergleich zu Radfahrern kann außerdem darauf geschlossen werden, dass sich die individuellen körperlichen Belastungen bei verschiedenen Sportarten bezüglich des Effekts auf den Knochenmetabolismus unterscheiden können [28]. Hier wird davon ausgegangen, dass die auftretenden Maximalkräfte maßgeblich für einen osteogenen Stimulus sind, was sich in erhöhten Knochenformationsmarkern äußert [28, 29].

Ein bewährter Knochenresorptionsmarker, der die Osteoklastenaktivität widerspiegelt, stellt die Kollagenquervernetzung („Crosslink“) Desoxypyridinolin (DPD) dar. DPD fungiert als molekulare Brücke der Extrazellulärmatrix zwischen Kollagenmolekülen des Typs 1 und ist nahezu ausschließlich im Knochen und Dentin anzutreffen. Nach resorptiver Wirkung der Osteoklasten resultiert eine vermehrte Abgabe der Crosslinks an Blut und Urin, wo sie quantitativ bestimmt werden können. Goldstandard ist die Bestimmung der DPD-Konzentration im Urin. Erhöhte DPD-Konzentrationen stehen somit mit einer katabolen Knochenstoffwechsellage und einem erhöhten Risiko für Knochenfrakturen im direkten Zusammenhang und können über die Reduktion der Knochenmineraldichte mit der Entstehung einer Osteoporose assoziiert sein [24, 30]. Generell gilt es zu beachten, dass Jugendliche höhere Formations- und Resorptionsmarker aufgrund des gesteigerten Knochenumbaus während des Wachstums aufweisen [31].

## Muskelstoffwechsel

Die Erfassung des Muskelstoffwechsels ist im funktionellen Assessment respektive der Leistungsdiagnostik von Athleten ein wichtiger Bestandteil. Neben direkten Traumata können sich auch intensive körperliche Belastungen bei Ungleichgewicht zwischen Belastung und Trainingsadaptation in Mikro- oder Makroschädigungen in den Muskeln äußern. Mittels Serummarker wie der Laktatdehydrogenase (LDH), der Kreatinkinase (CK), dem Myoglobin und der Aspartat-Aminotransferase (ASAT = GOT) lässt sich ein Monitoring der metabolischen Adaptation an das physische Training durchführen und es lassen sich Aussagen über die muskuläre Arbeitslast oder mögliche Schädigungen gewinnen [32]. Die messbaren Enzymaktivitäten zeigen eine direkte Korrelation zu der Belastungsintensität und können bis zu dem 4fachen des Ausgangswertes ansteigen [32]. Eine medikamenteninduzierte Erhöhung (z. B. CK-Erhöhung bei Statinen oder Steroiden) muss bei der Interpretation mitberücksichtigt werden [33].

Die CK katalysiert die Phosphorylierung von Adenosindiphosphat (ADP) zu Adenosintriphosphat (ATP) und besitzt daher eine zentrale Rolle im Energiestoffwechsel. Isoenzyme sind in verschiedenen Organen vorzufinden: CK-MM im Skelettmuskel, CK-MB im Herzmuskel und CK-BB im Gehirn. Unter physiologischen Umständen ist ausschließlich CK-MM im Blutserum nachzuweisen. Das Vorkommen anderer Isoenzyme sollte als verdächtig betrachtet werden. Zwar wurde unter anderem ein Nachweis von CK-MB bei Ultramarathonläufern und CK-BB bei Boxern beobachtet [33, 34], allerdings sollten diese Nachweise immer kritisch hinterfragt werden. Ebenfalls sollte bei Vorliegen einer erhöhten Gesamt-CK-Aktivität in Ruhephasen, auch bei Fehlen von prädisponierenden Faktoren, eine Diagnostik samt kardialer Labordiagnostik und Echokardiografie erfolgen. Zu beachten ist, dass Sportler physiologisch höhere CK-Aktivitäten besitzen als sportlich inaktive Menschen [33, 35]. So empfahlen Meyer et al. nach Analyse von 467 männlichen Profifußballspielern der 1. und 2. Bundesliga fußballspezifische Referenzbereiche für die CK. Grund für erhöhte CK-Werte scheinen die fußballspezifischen Bewegungen mit ihrem Stop-and-go-Charakter zu sein, welche zu einer hohen exzentrischen Belastung führen und eine stärkere Freisetzung der CK aus dem Zytosol der Muskelzellen bedingen [35]. Da generell bei den meisten Athleten nach körperlichen Belastungen ein Anstieg der CK zu verzeichnen ist, wird sie zum Nachweis von Skelettmuskelschäden bei Sportlern selten verwendet. Nach körperlicher Belastung sind bei einigen Athleten aufgrund der Trainingsadaptation geringere respektive sogar kaum vorhandene Anstiege der CK-Aktivität zu verzeichnen, man spricht hier von Non-Respondern [33, 36].

Ein Performance-Test mit maximaler Ausbelastung des Athleten kann für eine Evaluation der Variabilität der individuellen CK-Werte von Nutzen sein. Messungen vor der Belastung und 30 min, 6 h, 24 h, 48 h, und 72 h nach der körperlichen Anstrengung scheinen sinnvoll, um den dynamischen Verlauf darzustellen. Ein Peak der CK-Aktivität nach 24 h ist zu erwarten, während eine Normalisierung nach 48–72 h folgen sollte [33]. Unterschiede der physischen Belastungscharakteristika zwischen Kraft- und Ausdauerathleten sind bei der Interpretation zu beachten. So lassen sich bei Kraftathleten, vor allem nach Ausübung von exzentrischem Krafttraining, hohe CK-Aktivitäten nachweisen [37, 38].

Die LDH, ein Enzym aus der Gruppe der Oxidoreduktasen, wandelt, unter Umwandlung von NAD^+^ und seiner reduzierten Form NADH, Pyruvat und Laktat ineinander um. Nach körperlicher Belastung beziehungsweise muskulären Verletzungen resultieren im Vergleich zur CK langsamer steigende LDH-Aktivitäten, welche nach ausdauernder körperlicher Aktivität für 14 Tage erhöht sein können [39]. Der Anstieg geschieht vor allem am dritten bis fünften Tag nach dem Reiz [32]. Während sich nicht trainierte Menschen bezüglich der LDH-Konzentration in Ruhe nicht von trainierten Athleten unterscheiden, konnte bei diesen bereits nach einem 300-m-Sprint im Vergleich zu Athleten signifikant höhere LDH-Konzentrationen bestimmt werden [40]. Dieses lässt eine schnellere Schädigung des Muskelgewebes durch eine fehlende Trainingsadaptation vermuten und unterstreicht die Notwendigkeit eines guten Trainingszustandes, um Muskelverletzungen präventiv entgegenzuwirken.

Das seit vielen Jahren im Breiten- sowie im Spitzensport als diagnostisches Tool zur Leistungsdiagnostik und Trainingssteuerung verwendete Laktat entsteht bei hohen körperlichen Belastungen aus Pyruvat, welches während der anaeroben Glykolyse mithilfe der Laktatdehydrogenase in Laktat umgewandelt wird. Kurz zusammengefasst steigt mit verbessertem Trainingszustand die Laktatkonzentration erst bei einer höheren Belastung an. Somit spiegelt die Laktatkonzentration im Blut die kurzfristige (innerhalb von Minuten) metabolische Beanspruchung wider, wobei das Konzept der Laktatschwellen den Übergang von einer aeroben zur anaeroben Energiebereitstellung umfasst [41]. Die in standardisierten Belastungsprotokollen gemessenen Laktatwerte stellen in Form von Laktatleistungskurven eine seit vielen Jahren genutzte, unverzichtbare Hilfe in der leistungsorientierten Trainingssteuerung dar, obwohl dieses diagnostische Mittel auch einige Schwächen offenbart, wie die Abhängigkeit von anderen Faktoren wie der Ernährung oder der Vorbelastung [42].

Das zytoplasmatische Hämoprotein Myoglobin, welches aus einer Polypeptidkette und einem Porphyrinring mit zentralem Eisenmolekül besteht, stellt das sauerstoffbindende Protein des Muskels dar. Es wird ausschließlich in den Herzmuskelzellen und in oxidativen Skelettmuskelfasern exprimiert und ist fähig, Sauerstoff (O_2_) reversibel zu binden. Es ist in der Lage, bei Vorliegen einer Hypoxie Sauerstoff der Oxidation zur Verfügung zu stellen. Nach anstrengender körperlicher Betätigung kommt es durch den Abbau von Muskelproteinen zu einer Freisetzung von Myoglobin, welches bereits nach 30 min messbar ist [43]. Eine erhöhte Myoglobinkonzentration kann für 5 Tage verbleiben, vermutlich aufgrund moderater Inflammationsprozesse [32]. So ist eine Korrelation der Aktivitäten von CK und Myoglobin mit der durch Stress induzierten Reaktion der neutrophilen Granulozyten bekannt, wobei eine ausreichende Proteinzufuhr eine Abschwächung des Anstiegs bewirken kann [32]. Im Blut wird es hauptsächlich – neben anderen Parametern – zum Ausschluss eines kardialen Geschehens genutzt. Als weiterer Serummarker für Muskelschädigungen kann die Transaminase ASAT angesehen werden, für welche Meyer und Kollegen ebenfalls einen fußball- und mannschaftssportspezifischen Referenzbereich empfehlen [35]. Die ASAT stellt hierbei im Gegensatz zur leberspezifischen Transaminase ALAT (Alanin-Aminotransferase = GPT) eine ubiquitär und in großem Maße in den Muskelzellen vorkommende Transaminase dar.

Neben der Leistungsdiagnostik bietet das laborchemische Assessment mit den daraus abgeleiteten Handlungsempfehlungen weitere Möglichkeiten, die muskuläre Leistungsfähigkeit des Athleten zu optimieren, wie zum Beispiel durch ein Monitoring des Vitamin-D-Spiegels. Ein ausgeglichener Vitamin-D-Haushalt stellt eine wichtige Voraussetzung für die muskuläre Leistungsfähigkeit dar. Bei 61 männlichen britischen Athleten verschiedener Sportarten (Rugby, Fußball und professionelle Pferderennreiter) konnte durch die Optimierung des Vitamin-D-Status bereits nach einer 8‑wöchigen Supplementation von 5000 IE Vitamin D pro Tag eine signifikante Verbesserung der 10-m-Sprintzeit und der vertikalen Sprungfähigkeit festgestellt werden [44]. Bei 24 professionellen Balletttänzern und -tänzerinnen, bei denen initial Vitamin-D-Spiegel von < 30 ng/ml vorlagen, konnte dieses bestätigt werden: Nach einer 4‑monatigen Substitution von 2000 IE Vitamin D pro Tag wurde neben einer Verbesserung der Sprungkraft um 7,1 % außerdem eine signifikante Steigerung der isometrischen Kraft des M. quadriceps femoris um 18,7 % beobachtet [20]. So scheint ein Vitamin-D-Serumwert von > 40 ng/ml die Muskelkraft und -funktion, vor allem bei Athleten mit schnellkraftbetonten Sportarten, signifikant zu verbessern [45]. Außerdem konnte bei Typ-II-Muskelfasern („fast twitch fibres“), welche essenziell für sportliche Höchstleistung und für die Vermeidung von Stürzen sind, bei Vitamin-D-Mangelzuständen Muskelfaseratrophien mit Fettinfiltrationen und Fibrosen beobachtet werden, die nach Supplementierung partiell reversibel waren [22, 46].

Auch ein Zusammenhang zwischen posttraumatischem und altersbedingtem Muskelabbau und Vitamin D wird vermutet [47]. Weitere Untersuchungen konnten zeigen, dass Vitamin D nicht nur bei der muskulären Zelldifferenzierung, sondern auch bei der Zellproliferation bzw. der Proteinbiosynthese im mitochondrialen Metabolismus der Zellen eine wichtige Rolle spielt. Dies ist unter anderen durch eine Erhöhung des oxidativen Stresses und einer Reduktion der Sauerstoffverbrauchsrate in der Skelettmuskulatur bei Vitamin-D-Mangel zu begründen, wobei die molekularen Mechanismen komplex und zum Teil unerforscht sind [48]. Schlussendlich konnte ein systematisches Review positive Auswirkungen von ausgeglichenen Vitamin-D-Spiegeln auf die Muskelkraft demonstrieren, obwohl eine hohe Variabilität bezüglich der Effektstärken besteht [49]. Bezüglich des Zusammenhangs von niedrigen Vitamin-D-Spiegeln und akuten Muskelverletzungen scheint es hingegen deutlich weniger Evidenz zu geben. Eine Auswahl der geeigneten laborchemischen Parameter der muskuloskelettalen Labordiagnostik ist in der Tab. 1 dargestellt.

**Table 1:** 

Parameter	Bedeutung	Abweichung
Calcium^a^	Mineralstoff der Skelettmineralisation	↓ Höhere Inzidenz für Stressfrakturen [16]; chronischer Mangel kann in einer Osteomalazie resultieren
Phosphat^a^	Mineralstoff der Skelettmineralisation	↓ Chronischer Mangel kann in einer hypophosphatämischen Osteomalazie resultieren [6]
Vitamin D (25(OH)D_3_)^a^	Schlüsselfunktion in der Calciumhomöostase und Skelettmineralisation	↑ ≥ 40 ng/ml präventiver Nutzen bezüglich (Stress‑)Frakturen [22] ≥50 ng/ml optimale Voraussetzung für maximale Leistungsfähigkeit [22] ↓ < 30 ng/ml reduzierte Calciumresorption; höhere Inzidenz für Stressfrakturen [14, 15] und „delayed union“ [23]
Osteocalcin (Oc)^a^	Calciumbindendes Peptidhormon der Osteoblasten; Knochenformationsmarker	↓ Kataboler/„low-turnover“ Knochenstoffwechsel [24, 28]
Knochenspezifische AP (BAP)^a^	Enzym der Knochenformation; Knochenformationsmarker	↑ Bei Dissoziation zu Osteocalcin (Verhältnis BAP>Oc): Hinweis auf osteomalazische Komponente ↓ Kataboler/„low-turnover“ Knochenstoffwechsel [24], Hypophosphatasie
Desoxypyridinolin (DPD)^a^	Produkt der Knochenresorption; Knochenresorptionsmarker	↑ Erhöhte Knochenresorption mit gesteigertem Risiko für Frakturen [24]
Parathormon (PTH)^a^	Regulationsfunktion in der Calciumhomöostase	↑ Primärer/tertiärer Hyperparathyreoidismus: Calcium (↑), Phosphat (↓); sekundärer Hyperparathyreoidismus: Calcium (↓/*n*), Phosphat (↑/*n*) [9, 12]
Kreatinkinase (CK)^b^	Energiebereitstellung durch Rephosphorylierung von ADP zu ATP	↑ Muskelzellschädigung; medikamenteninduziert (beispielsweise Statine, Steroide) [33]; potenzielles Übertraining [76]
Laktatdehydrogenase (LDH)^b^	Energiebereitstellung im anaeroben Energiestoffwechsel	↑ Muskelzellschädigung [39]
Aspartat-Aminotransferase (ASAT)^b^	Transaminase mit ubiquitärem Vorkommen	↑ Muskel- und Leberzellschädigung durch körperliche Belastung [77]
Laktat^b^	Endprodukt der anaeroben Glykolyse	↑ Akute körperliche Belastung; potenzielles Übertraining [76]
Magnesium (Mg)	Mineralstoff mit Einfluss auf die Skelettmineralisation	↑ Aktivierung der Osteoklasten [67] ↓ Inhibierung der Osteoblasten und Aktivierung der Osteoklasten [67]; Verringerung der muskulären Leistungsfähigkeit und Integrität [68]
Eisen, Ferritin (Fe)	Mineralstoff mit Einfluss auf die Blutbildung und Leistungsfähigkeit	↓ Erhöhtes Risiko für Frakturen und verlängerte Regenerationsdauer nach Verletzungen [50, 61]
Zink (Zn)	Mineralstoff mit Einfluss auf die Skelettmineralisation	↓ Negativer Effekt auf die Skelettmineralisation mit möglichem reduziertem Knochenmineralsalzgehalt [70]

Die dargestellten Parameter stellen lediglich eine Auswahl dar. Vor allem im Kapitel Nährstoffe wird ebenfalls auf die Relevanz weiterer Makro- und Mikronährstoffe (Proteine, Vitamine wie Vitamin B_6_, B_9_ und B_12_) hingewiesen. ^a^Calcium- und Knochenstoffwechsel, ^b^Muskelstoffwechsel

## Nährstoffe, Energieverfügbarkeit und Vitamine

Eine angemessene körperliche Bewegung und Belastung gilt generell als osteoprotektiv. So konnte bei Athletengruppen aus Sportarten mit hohen Maximalkräften und multidirektionalen Bewegungen, wie z. B. Fußball, Volleyball oder auch Rugby, eine bessere Knochenqualität festgestellt werden, während Athleten aus Ausdauersportarten mit niedrigen Maximalkräften und niedriger Energieverfügbarkeit, wie Langstreckenlauf, Schwimmen oder Radrennen, eine reduzierte Knochenmasse aufwiesen [50]. Da 90 % der Gesamtknochenmasse bis zum 20. Lebensjahr generiert werden und der Aufbau mit dem 30. Lebensjahr weitgehend abgeschlossen ist („peak bone mass“) [50], ist anzustreben, dass trotz einer aktiven Sportlerkarriere in diesen Jahren suffiziente Bedingungen für einen Knochenmasseaufbau geboten werden. Als positiver Stimulus für den Knochenstoffwechsel wird der mechanischen Belastung der Knochen mit dem Auftreten von Maximalkräften eine entscheidende Rolle beigemessen [51]. Dabei stellt jedoch ein weiterer wichtiger Einflussfaktor die Ernährung respektive die Energieverfügbarkeit dar.

Ihle und Loucks (2004) untersuchten die Dosis-Wirkungs-Beziehung zwischen drei Stufen verminderter Energieverfügbarkeit und dem Knochenstoffwechsel von jungen gesunden Frauen im Vergleich zu energiebalancierten Kontrollen mit einer Energieverfügbarkeit von 45 kcal/kg fettfreier Körpermasse („lean body mass“ [LBM])/Tag. So waren bei Energieverfügbarkeiten von 30 beziehungsweise 20 kcal/kg LBM/Tag signifikant geringere Knochenformationsraten zu detektieren, während die Knochenresorption noch unverändert blieb. Bei weiterer Reduktion der Energieverfügbarkeit auf 10 kcal/kg LBM/Tag stieg zusätzlich die Knochenresorption an, mit der Gefahr einer katabolen Knochenstoffwechsellage [52]. Hier gilt es zu beachten, dass neben einer geringeren Energieaufnahme auch ein hoher Energieverbrauch bei Athleten eine geringere Energiebilanz bedingt, was vor allem für Ausdauerathleten relevant ist. So werden Eliteausdauerathleten das Niveau von 45 kcal/kg LBM/Tag angesichts der hohen Energieausgaben nur schwerlich erreichen können [53]. Außerdem sehen viele Ausdauersportler ein Energiedefizit als essenziell an, um den Phänotyp des Ausdauerathleten mit einem möglichst großen Anteil fettfreier Masse zu generieren. Nichtsdestotrotz ist eine ausreichende Energieverfügbarkeit beziehungsweise suffiziente Versorgung mit Nährstoffen anzustreben, um die kurz- und langfristige Knochengesundheit zu gewährleisten.

Die Kombination aus einer geringen Energieverfügbarkeit (mit oder ohne Essstörung), einem veränderten Menstruationszyklus mit niedrigeren Östrogenen und anderen Hormonstörungen und einer verminderten Knochenmineraldichte („bone mineral density“ [BMD]) beschreibt einen Zustand, der vor allem bei intensiv sporttreibenden Frauen beobachtet wird und vormals als „female athlete triad“ bezeichnet wurde [54]. Da mittlerweile bekannt ist, dass der relative Energiemangel die Grundproblematik darstellt und auch Männer betroffen sein können, wurde die Terminologie in „relative energy deficiency in sport“ (RED-S) geändert [55]. Der Begriff stellt eine Erweiterung der Triade der weiblichen Athletin dar, scheint jedoch im Bewusstsein der Ärztinnen und Ärzte noch nicht ausreichend vertreten zu sein [56]. Neben der langfristigen Verringerung der Knochenmineraldichte wurde in diesem Zusammenhang auch eine höhere Inzidenz von Stressfrakturen beschrieben [57].

Als Orientierung für entsprechende Mangelzustände kann neben einer Bilanzierung der Energieverfügbarkeit eine labordiagnostische Bestimmung von Nährstoffen dienen, um eine Limitation der Entwicklung und Funktionstüchtigkeit des muskuloskelettalen Systems durch das Vorliegen von Mangelzuständen auszuschließen. Das Erkennen und Beseitigen von Mangelzuständen stellt eine Grundvoraussetzung für die Optimierung der Leistungs- und Regenerationsfähigkeit der Athleten dar. Calcium, Vitamin D und Phosphat wurden als maßgebliche osteologische Parameter bereits angeführt. Weitere Parameter stellen als Makronährstoffe die Proteine und als Mikronährstoffe Eisen, Magnesium und Zink sowie Vitamin B_6_, B_9_ und B_12_ dar. Die von der International Society of Sports Nutrition (ISSN) empfohlene tägliche Proteinzufuhr für gesunde Sportler liegt bei 1,4–2,0 g Protein/kg Körpergewicht, um durch eine positive Proteinbilanz bestmöglich den Muskelaufbau/-erhalt und die Trainingsadaptation zu garantieren, während einem Muskelverlust vorgebeugt werden soll [58]. Proteine als Bestandteil des Kollagens und der Wachstumsfaktoren haben folglich ebenfalls einen positiven Effekt auf den Knochen [50]. Für eine Beurteilung der individuell benötigten Menge an Proteinen pro Tag können unterstützend Biomarker herangezogen werden (Gesamtprotein, Albumin, Stickstoffbilanz, Harnstoff-Stickstoff, Aminosäurenanalyse). Eine Fehlernährung mit resultierendem Proteinmangel scheint hierbei mit einer Reduktion der Albuminsynthese und einem verminderten Gesamtprotein einherzugehen. Zusätzlich kann die Stickstoffbilanz, zum Beispiel durch eine Messung des Harnstoff-Stickstoffs als Abbauprodukt von Proteinen (im Blut oder Urin) Aufschlüsse über den Eiweißstoffwechsel liefern und beispielsweise auf Mangelernährung hinweisen [59].

Ein Eisenmangel stellt einen leistungslimitierenden Faktor dar, der sich laborchemisch in Form eines erniedrigten Ferritins beziehungsweise Hämoglobins im Falle einer Eisenmangelanämie widerspiegelt [60] und sich klinisch unter anderem durch eine Ermüdung („Fatigue“) mit verringerter maximaler Sauerstoffaufnahme (VO_2max_) und Dyspnoe äußert [61]. Auf muskuloskelettaler Ebene kann sich ein Eisenmangel in einem erhöhten Risiko für eine herabgesetzte Knochendichte, für Stressfrakturen, oder aber auch durch eine verlängerte Regenerationsdauer nach Verletzungen manifestieren [50, 61–63]. Bei 1085 Eliteathleten (570 weiblich, 515 männlich) aus über 26 Sportarten konnte bei 15 % der männlichen und 52 % der weiblichen Athleten ein defizitärer Eisenstatus festgestellt werden [64]. Als möglicher Einflussfaktor der deutlich höheren Prävalenz eines Eisenmangels bei Athletinnen ist hier ein menstruationsbedingter Eisenverlust zu bedenken [65].

Magnesium (Mg) beeinflusst als Cofaktor zahlreicher anaboler und kataboler Reaktionen ebenfalls die Knochengesundheit und Muskelleistung des Athleten [66], wobei niedrige Magnesiumkonzentrationen möglicherweise eine katabole Stoffwechselsituation des Knochens herbeiführen können [67]. Ein positiver Zusammenhang zwischen Magnesiumstatus, der Muskelkraft und der Muskelleistung durch eine muskelprotektive Wirkung respektive Aufrechterhaltung der Muskelintegrität ist ebenfalls bekannt [68]. So konnten bei Radsportlern nach Magnesiumsupplementation (400 mg/d) während eines 21-tägigen Etappenrennens signifikant niedrigere Myoglobinkonzentrationen nachgewiesen werden [69]. Zink (Zn) ist ein weiterer Mineralstoff, der aufgrund des erhöhten Bedarfs bei Sportlern abgedeckt sein sollte und für eine Vielzahl von Funktionen der Wundheilung, der Glukoseverwertung und der Proteinsynthese erforderlich ist und sowohl antioxidativ als auch antiinflammatorisch wirkt [59]. Daneben nimmt Zink als Cofaktor mehrerer Enzyme, wie der AP und der Kollagenase, eine Rolle in der Knochenmineralisation und der Synthese der kollagenen Strukturen des Knochens ein [70]. Ein Zinkmangel ist kein seltenes Ereignis und kann durch eine suboptimale Zinkaufnahme bei körperlichen Belastungen, Stress und einseitigen Ernährungsgewohnheiten bedingt sein und zu einer verminderten Knochendichte führen [70, 71].

Vitamine stellen eine Gruppe von lebensnotwendigen organischen Verbindungen unterschiedlicher Stoffklassen dar, welche vom Körper nicht selbst synthetisiert werden können und durch die Nahrung aufgenommen werden müssen. Trotz der Wichtigkeit einer adäquaten Aufnahme der Vitamine C, E und K ist die Verlässlichkeit und der Nutzen ihrer laborchemischen Bestimmung nicht vollständig nachgewiesen und wird in dieser Arbeit nicht thematisiert. Für das labordiagnostische Assessment sind vor allem die Vitamine B_6_, B_9_ und B_12_ geeignet. Der Vitamin-B-Komplex spielt eine bedeutende Rolle in der Regulation des Energiemetabolismus [59]. Wichtige Vertreter stellen das B_6_ (Pyridoxin), B_9_ (Folsäure) und B_12_ (Cobalamin) dar. So hat Pyridoxin eine zentrale Stellung als Coenzym vieler Reaktionen des Aminosäurenstoffwechsels und trägt zur Synthese von Fettsäuren bei [72]. Folsäure ist maßgeblich an Wachstums- und Zellteilungsprozessen im menschlichen Körper beteiligt [73]. Eine Analyse des Cobalamins, welches vorwiegend in tierischen Produkten vorkommt, ist vor allem Vegetariern und Veganern empfohlen und es sollte gegebenenfalls substituiert werden. Ein Cobalaminmangel scheint mit einer Verminderung der Knochendichte sowie einer Beeinträchtigung der Osteoblastenfunktion einherzugehen [74, 75].

## Periodisierung des laborchemischen Assessments

**Figure 3:**
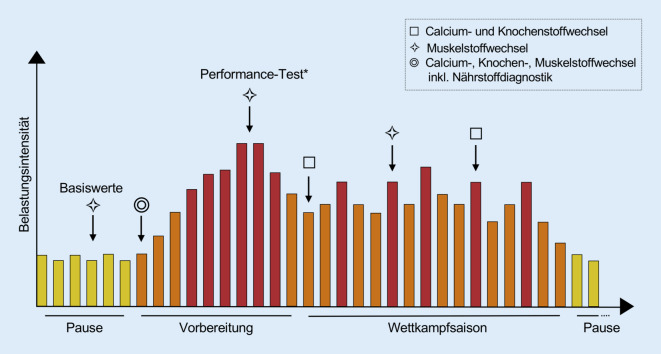

Ein individuelles Vorgehen im Rahmen der Labordiagnostik erscheint sinnvoll, da individuell zwischen Athleten bedeutende Unterschiede der labordiagnostischen Referenzintervalle auftreten. So sind bedeutende belastungsabhängige und sportartspezifische Unterschiede zahlreicher Laborparameter zwischen Athleten nachzuweisen [35, 59]. Es ist sinnvoll, aber zeitlich und technisch aufwändig, durch eine Periodisierung der Messungen personalisierte Referenz- und Variabilitätsbereiche der Athleten zu bestimmen, welche im Rahmen von unterschiedlichen Trainings- und Belastungszuständen durchgeführt werden (Abb. 3). Es wird jedoch auch deutlich, dass keine pauschale Aussage über die Zeitintervalle zwischen den Laboranalysen sinnvoll ist, sondern ein bedarfs- und saisonabhängiges Vorgehen gewählt werden sollte. Ein Mindestmaß von zwei Labordiagnostiken pro Jahr ist jedoch zu empfehlen, um die medizinische Grundversorgung eines Leistungssportlers gewährleisten zu können.

Auch während der Rehabilitation nach einer Verletzung können zuvor erhobene Basiswerte der individuellen Referenz- und Variabilitätsbereiche helfen, Verlaufswerte besser einzuschätzen.

## Fazit für die Praxis

- Ein regelmäßig durchgeführtes labordiagnostisches Assessment (im Leistungssport zumindest zweimal pro Jahr) und die daraus abgeleitete Intervention bei auffälligen Werten kann helfen, die Leistungsfähigkeit von Athleten zu verbessern.
- Eine ausgeglichene Calciumhomöostase sollte durch eine optimale Vitamin-D-Versorgung sowie eine ausgewogene Ernährung angestrebt werden, um das Risiko von Verletzungen wie Stressfrakturen zu verringern.
- Das laborchemische Assessment beinhaltet verschiedene Muskelenzyme, sowie Makro- und Mikronährstoffe unter Berücksichtigung des individuellen Energiebedarfs.
- Allgemeine Referenzbereiche dienen als Orientierung, wobei bei Leistungssportlern ein individuelles Vorgehen mit Aufstellung individueller und ggf. sportartspezifischer Referenzbereiche sinnvoll erscheint.
- Eine detaillierte laborchemische Diagnostik sollte heutzutage fester Bestandteil in der ärztlichen Betreuung von professionellen Athleten.

## Abkürzungen

ADP: Adenosindiphosphat
ALAT: Alanin-Aminotransferase
AP: Alkalische Phosphatase
ASAT: Aspartat-Aminotransferase
ATP: Adenosintriphosphat
BAP: „Bone-specific alkaline phosphatase“
BMD: „Bone mineral density“
CK: Kreatinkinase
DPD: Desoxypyridinolin
FGF: „Fibroblast growth factor“
ISSN: „International Society of Sports Nutrition“
LBM: „Lean body mass“
LDH: Laktatdehydrogenase
NAD: Nicotinamidadenindinukleotid
NFL: National Football League
Oc: Osteocalcin
PTH: Parathormon
RED‑S: „Relative energy deficiency in sport“
